# Priority in Selenium Homeostasis Involves Regulation of SepSecS Transcription in the Chicken Brain

**DOI:** 10.1371/journal.pone.0035761

**Published:** 2012-04-20

**Authors:** Jin-Long Li, Hui-Xin Li, Xue-Jiao Gao, Jiu-Li Zhang, Shu Li, Shi-Wen Xu, Zhao-Xin Tang

**Affiliations:** 1 College of Veterinary Medicine, South China Agricultural University, Guangzhou, People's Republic of China; 2 College of Veterinary Medicine, Northeast Agricultural University, Harbin, People's Republic of China; 3 Division of Avian Infectious Diseases, State Key Laboratory of Veterinary Biotechnology, Harbin Veterinary Research Institute, Chinese Academy of Agricultural Sciences, Harbin, People's Republic of China; Trinity College, Ireland

## Abstract

O-Phosphoseryl-tRNA:selenocysteinyl-tRNA synthase (SepSecS) is critical for the biosynthesis and transformation of selenocysteine (Sec) and plays an important role in the biological function of Se through the regulation of selenoprotein synthesis. Selenium (Se) and Selenoprotein play a pivotal role in brain function. However, how intake of the micronutrient Se affects gene expression and how genetic factors influence Se metabolism in the brain is unknown. To investigate the regulation of SepSecS transcription induced by Se in the chicken brain, we determined the Se content of brain tissue, SepSecS gene expression levels and mRNA stability in the chicken brain and primary cultured chicken embryos neurons receiving Se supplements. These results showed that Se content in the brain remains remarkably stable during Se supplementation. A significant increase in SepSecS mRNA levels was observed in all of the brain tissues of chickens fed diets containing 1–5 mg/kg sodium selenite. Most strikingly, significant changes in SepSecS mRNA levels were not observed in neurons treated with Se. However, Se altered the SepSecS mRNA half-life in cells. These data suggest that Se could regulate SepSecS mRNA stability in the avian brain and that SepSecS plays an important role in Se homeostasis regulation.

## Introduction

Selenium (Se) is an essential micronutrient in the diet of many life forms, including birds and mammals. Significant health benefits have been attributed to this element. In addition to the well-documented functions of Se as an antioxidant, in thyroid regulation, immune function [1], and in chemoprevention [2]–[3], previous data suggest that Se plays an important roles in brain function [4]–[6]. Evidence for this comes from the remarkable stability of brain Se levels during times of dietary Se restriction in experimental animals [3], [5]–[6]. Se is widely distributed throughout the body, but Se levels in the brain are not high compared with most other organs, remains remarkably stable during Se deficiencies, most likely at the expense of other organs [4], [7]–[8]. Additional evidence for the brain being at the apex of Se retention is provided by a study showing that a six generation Se deficiency in rats caused a more than 99% reduction of Se concentration in the liver, blood, skeletal tissue, and muscle, while the brain retained Se levels of 60% [9]. Similarly, the mRNA level of twelve selenoproteins in young pigs fed a Se-deficient diet remained high in the pituitary gland, but was significantly reduced in other organs such as the liver [10]. These results suggest that Se is critical for the maintenance of brain function, and is appropriated to the brain from the remaining parts of the body. The pattern of Se is found in many mammalian species including in rodents and primates. However, the effects of Se depletion in the avian brain are unknown.

**Figure 1:**
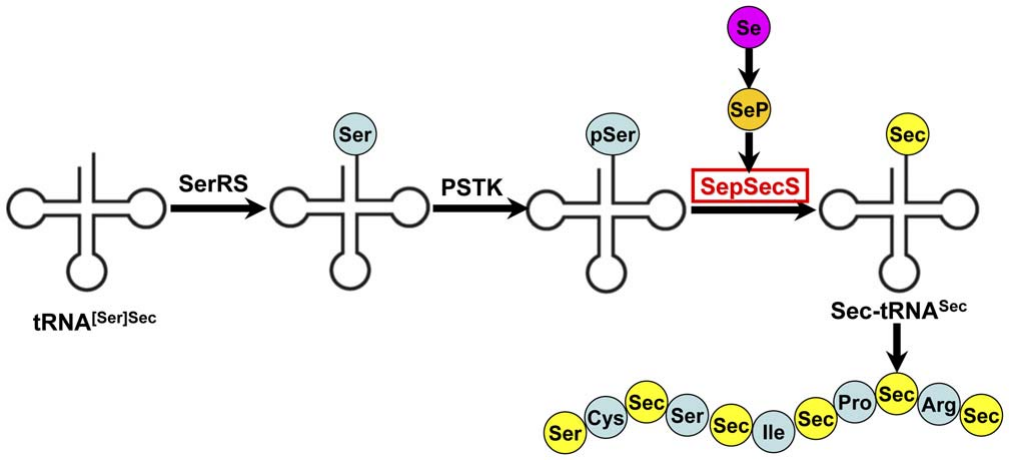
Sec biosynthesis and transformation in eukaryotes. The pathway of Sec biosynthesis and transformation is shown (see text for details; abbreviations are defined in the text) [12]–[14].

The molecular biology field has recently contributed significantly to the recognition of Se and selenoprotein as modulators of brain function [3], [6]. Nearly all Se in animal tissues is associated with protein. Several Se-binding proteins have been identified, but only one, selenocysteine (Sec) has been shown to be specific for and physiologically regulated by the element. Proteins containing this form of Se are referred to as selenoproteins. The biological function of Se is primarily implemented through its incorporation into selenoproteins. Sec is the most recently discovered eukaryotic amino acid encoded directly into proteins, making it the 21st amino acid. The synthesis of Sec and its insertion into polypeptides require a complex molecular machinery that recodes in-frame UGA codons, which normally function as stop signals, to serve as Sec codons [11]. The biosynthesis and transformation of Sec in eukaryotes is shown in Fig. 1. Sec is not attached directly to tRNA^Sec^, but is formed in a tRNA-dependent transformation of Ser to Sec. In the first step tRNA^[Ser]Sec^ is misacylated by seryl-tRNA synthetase (SerRS), an enzyme that could directly aminoacylate tRNA^Sec^ with Ser. The seryl moiety is phosphorylated by O-phosphoseryl-tRNA^[Ser]Sec^ kinase (PSTK) to form O-phosphoseryl-tRNA^[Ser]Sec^, and then the resulting tRNA-bound Sep is transformed to Sec by O-Phosphoseryl-tRNA:selenocysteinyl-tRNA synthase (SepSecS) in the presence of selenophosphate [12]–[14]. Then EFSec, the tRNA^Sec^-specific elongation factor, carries the Sec-tRNA^Sec^ to the ribosome where a translational recoding process allows UGA to be read as Sec [15]. This indicates that SepSecS is critical for the biosynthesis and transformation of Sec in eukaryotes, and plays an important role in the biological function of Se through the regulation of the selenoprotein synthesis.

The physiological functions of Se are thought to result from its existence in a number of selenoproteins in which Se is present, such as Sec. Previous studies suggest that selenoprotein synthesis is sensitive to the availability of Se *in vivo* or *in vitro* [16]–[20]. However, to our knowledge, the regulation of SepSecS gene expression by Se is not known. Herein, we used experimental analyses *in vivo* and *in vitro* to show that the stability of SepSecS mRNA in the chicken brain and primary cultured chick embryo neurons may be regulated by Se. Furthermore, we also demonstrated that Se content in the bird brain remains relatively stable when Se is supplemented.

## Results

### Se content in brain tissues

**Figure 2:**
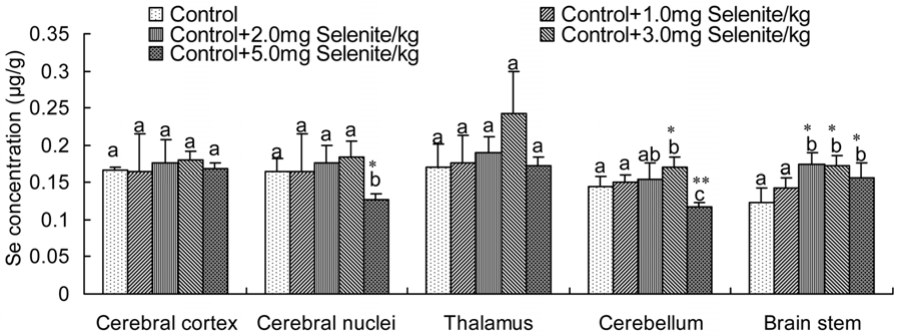
Brain tissue (µg/g, wet wt of tissue) Se content in chickens fed diets containing various concentrations of Se. Bars represent mean ± standard deviation (n = 3/group). Bars with “*” are significantly different from controls as measured by one-way analysis of variance followed by a Tukey's multiple comparison test (* *P*<0.05, ** *P*<0.01). Within the groups treated with various levels of Se, bars sharing a common letter (a or b or c) are not significantly different (*P*>0.05).

The effects of dietary Se concentrations on Se content in the brain are shown in Fig. 2. The Se content in the cerebrum and thalamus of chickens fed diets containing 1–5 mg/kg sodium selenite did not show a dose-dependent increase with increasing dietary Se concentrations. Chickens fed diets containing 1 mg/kg sodium selenite did not have significantly changed Se levels in the cerebellum and brain stem compared with chickens fed the basal diet. A significant increase in Se content of the brain stem was observed in chickens fed diets containing 2–5 mg/kg sodium selenite (*P*<0.01). Chickens fed diets containing 3 mg/kg sodium selenite also had a significant increase in Se in the cerebellum (*P*<0.05). Furthermore, there was a significant decrease in Se in the cerebellum of chickens fed diets containing 5 mg/kg sodium selenite, compared with chickens fed the basal diet (*P*<0.01).

### Effects of Se on the viability and morphology of neurons

**Figure 3:**
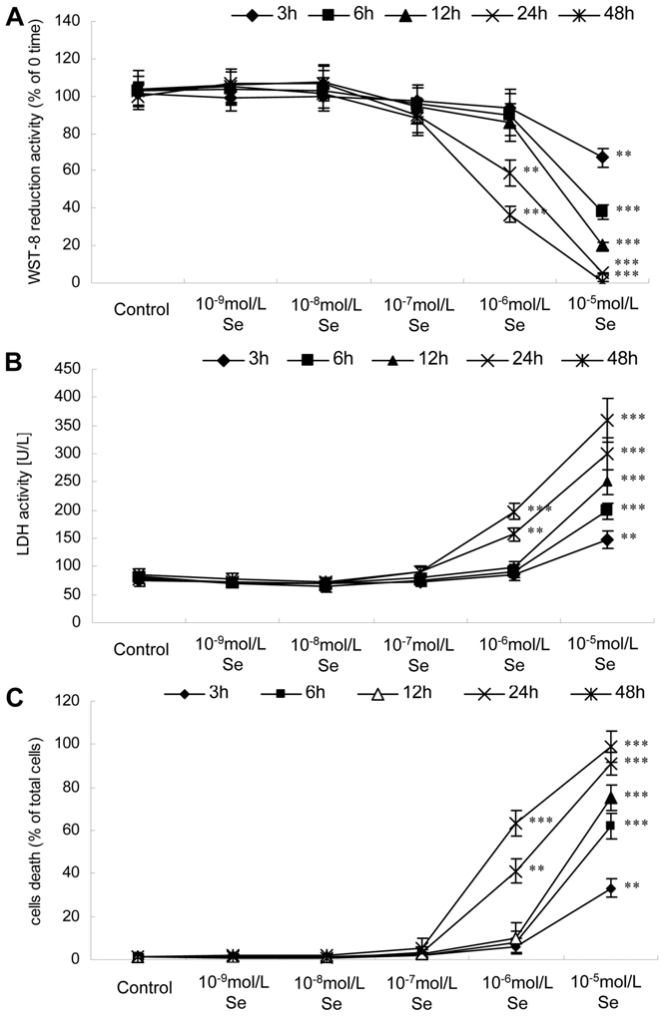
Effects of Se on neuron survival. The chicken embryo neuron monolayers were treated with 0 mol/L, 10^−9^ mol/L, 10^−8^ mol/L, 10^−7^ mol/L, 10^−6^ mol/L or 10^−5^ mol/L of Se as sodium selenite for 0 h, 3 h, 6 h, 12 h, 24 h or 48 h, respectively. The survival activity of neurons was measured by a WST-8 assay (A), and cell death was measured by a LDH release assay (B) and a trypan blue exclusion test (C). Cell viability was measured by a trypan blue exclusion test. Bars represent mean ± standard deviation (n = 3/group). Bars with “*” are significantly different from controls as measured by one-way analysis of variance followed by a Tukey's multiple comparison test (** *P*<0.01, *** *P*<0.001).

The relatively low concentration of Se (10^−9^–10^−7^ mol/L) had no effect on neuronal survival. In contrast, the high concentration of Se (10^−6^–10^−5^ mol/L) was neurotoxic to chick embryo neurons and significantly decreased cell viability. The survival of neurons treated with 10^−9^–10^−7^ mol/L of Se for 48 h and 10^−6^ mol/L of Se for 12 h did not change significantly (Fig. 3A). The decrease in WST-8 reduction activity was evident when neurons were treated with 10^−6^ mol/L of Se for 24 h and 10^−5^ mol/L of Se for 3 h (Fig. 3A). In experiments measuring death rates by an LDH activity assay, LDH activity remained remarkably stable in neurons treated with 10^−9^–10^−7^ mol/L of Se for 48 h and 10^−6^ mol/L of Se for 12 h, and increased in neurons treated with 10^−6^ mol/L of Se for 24 h and 10^−5^ mol/L of Se for 3 h (Fig. 3B). Similar results were observed when a trypan blue exclusion assay was used to measure cell death activity (Fig. 3C). In a quantitative analyses of neurons, relative low concentrations of Se (10^−9^–10^−7^ mol/L) enhanced neurite outgrowth (Fig. S1 and S2 A). High concentration of Se (10^−6^ mol/L) were neurotoxic to chick embryo neurons and significantly decreased neurite length and branches (Fig. S1 and S2 B–C).

### Effects of Se supplementation on SepSecS mRNA abundance

#### SepSecS mRNA abundance in the brain

**Figure 4:**
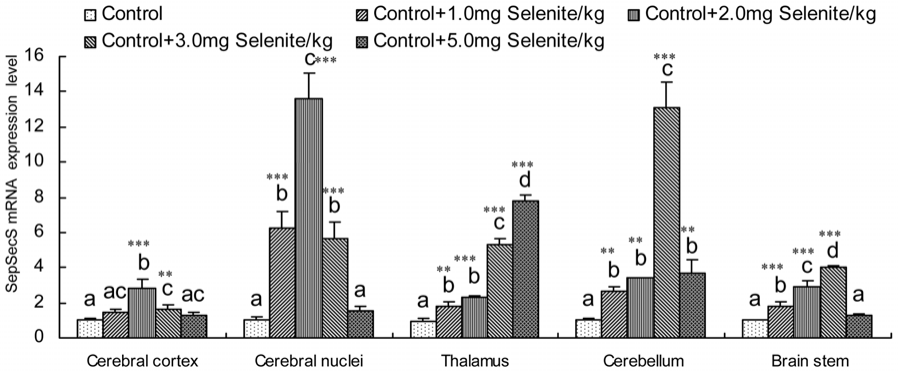
Effects of different concentrations of Se on the abundance of SepSecS mRNA in chicken brain tissue. Bars represent mean ± standard deviation (n = 3/group). Bars with “*” are significantly different from controls as measured by one-way analysis of variance followed by Tukey's multiple comparison test (* *P*<0.05, ** *P*<0.01, *** *P*<0.001). Within the groups treated with various levels of Se, bars sharing a common letter (a or b or c) are not significantly different (*P*>0.05).

SepSecS mRNA abundance in the brain measured by quantitative RT-PCR is shown in Fig. 4. A significant increase in SepSecS mRNA levels was observed in all brain tissues of chickens fed diets containing 1–5 mg/kg sodium selenite when compared with the control group. The greatest increases in SepSecS mRNA expression were observed in the cerebral cortex and cerebral nuclei of chickens fed diets containing 2 mg/kg sodium selenite (*P*<0.001), and in the cerebellum and brain stem of chickens fed diets containing 3 mg/kg sodium selenite (*P*<0.001). Of note, there was a dose-dependent increase in SepSecS mRNA expression in the thalamus with increasing dietary Se content when chickens were fed diets containing 1–5 mg/kg sodium selenite. However, after reaching a maximal level, further increases in Se doses actually led to a reduction of SepSecS mRNA abundance in the cerebral cortex, cerebral nuclei, cerebellum and brain stem (Fig. 4).

#### SepSecS mRNA abundance in the chick embryo neurons

**Figure 5:**
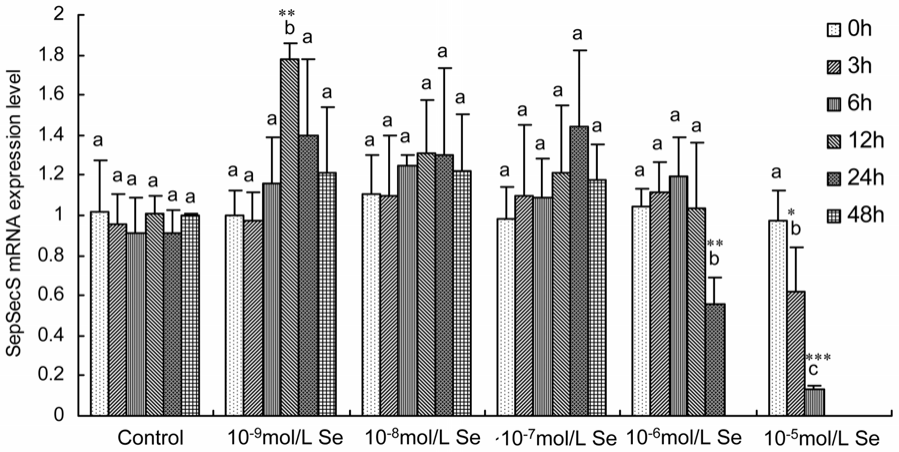
Effects of Se on SepSecS mRNA expression levels in chicken embryo neurons. The chicken embryo neuron monolayers were treated with 0 mol/L, 10^−9^ mol/L, 10^−8^ mol/L, 10^−7^ mol/L, 10^−6^ mol/L or 10^−5^ mol/L of Se as sodium selenite for 0 h, 3 h, 6 h, 12 h, 24 h or 48 h, respectively. SepSecS mRNA expression levels in chicken embryo neurons were measured by quantitative real-time RT-PCR, and the ratio of SepSecS mRNA to that of the GADPH internal control was used for statistical comparison. Bars represent mean ± standard deviation of triplicate cultures. Bars with “*” are significantly different from controls by one-way analysis of variance followed by a Tukey's multiple comparison test (* *P*<0.05, ** *P*<0.01, *** *P*<0.001). Within the groups treated with various concentrations of Se, bars sharing a common letter (a or b or c) are not significantly different (*P*>0.05).

SepSecS mRNA abundance in neurons remained remarkably stable when supplemented with relatively low Se concentrations, but a significantly decrease in SepSecS mRNA levels was found in cells treated with neurotoxic concentrations of Se. SepSecS mRNA levels in the neurons treated with 10^−9^–10^−6^ mol/L of Se for 0–48 h did not change significantly, except for 10^−9^ mol/L of Se for 12 h and 10^−6^ mol/L of Se for 48 h (*P*<0.01) when compared with the control group (Fig. 5). However, the SepSecS mRNA levels displayed a time-dependent decrease in neurons treated with 10^−5^ mol/L of Se, indicating that high concentrations of Se had cytotoxic effects in chick embryo neurons.

### Effect of Se supplementation on SepSecS mRNA stability

**Figure 6:**
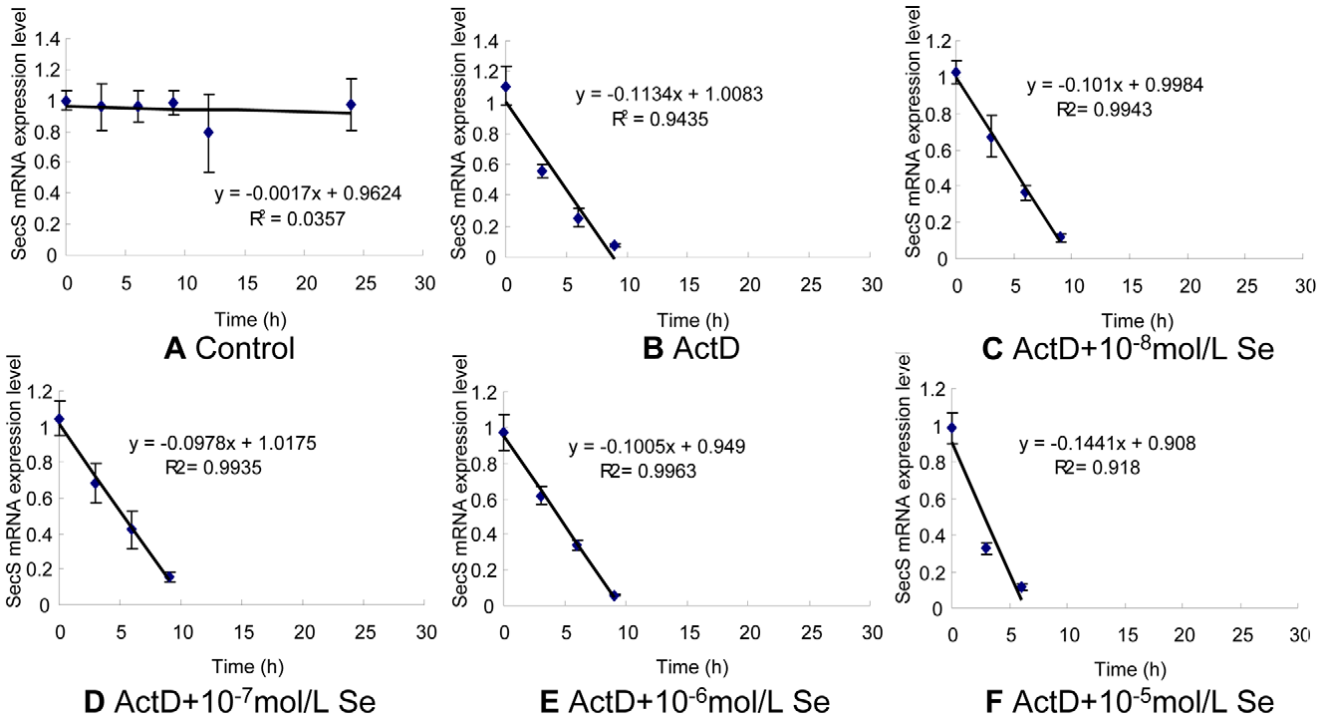
Effects of Se on the half-life of SepSecS mRNA in chicken embryo neurons. The chicken embryo neuron monolayers were treated with 0 mol/L, ActD, ActD+10^−8^ mol/L Se, ActD+10^−7^ mol/L Se, ActD+10^−6^ mol/L Se or ActD+10^−5^ mol/L Se for 0 h, 3 h, 6 h, 9 h, 12 h, 24 h or 48 h, respectively. SepSecS mRNA expression levels in chicken embryo neurons were measured by quantitative real-time RT-PCR. The RNA half-life was extrapolated from the SelW mRNA decay curve at the time point after 5 µg/mL ActD treatment.

**Figure 7:**
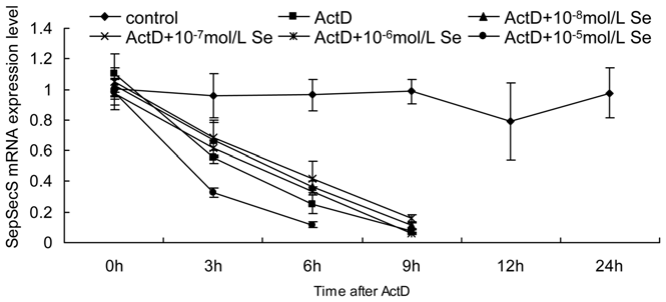
Effects of Se on SepSecS mRNA stability in chicken embryo neurons. The chicken embryo neuron monolayers were treated with 0 mol/L, ActD, ActD+10^−8^ mol/L Se, ActD+10^−7^ mol/L Se, ActD+10^−6^ mol/L Se or ActD+10^−5^ mol/L Se for 0 h, 3 h, 6 h, 9 h, 12 h, 24 h or 48 h, respectively. SepSecS mRNA expression levels in chicken embryo neurons were measured by quantitative real-time RT-PCR. The mRNA stability was denoted with the SepSecS mRNA decay curve after 5 µg/mL ActD treatment.

To examine the mechanisms by which Se influenced SepSecS mRNA, the SepSecS mRNA half-life in chick embryo neurons was measured in the presence or absence of actinomycin D (ActD). Cells were incubated with the RNA polymerase inhibitor ActD to block de novo mRNA synthesis and cells were harvested at indicated time points. The SepSecS mRNA half-life was observed in chick embryo neurons incubated with ActD (about 4.48 h), ActD+10^−8^ mol/L of Se (about 4.93 h), ActD+10^−7^ mol/L of Se (about 5.29 h), ActD+10^−6^ mol/L of Se (about 4.47 h) and ActD+10^−5^ mol/L of Se (about 2.83 h) (Fig. 6). Of note, in the presence of 10^−8^–10^−7^ mol/L Se, SepSecS mRNA levels were higher than in the presence of ActD (Fig. 6). However, the SepSecS mRNA levels of neurons treated with 10^−5^ mol/L Se was significantly reduced (Fig. 7). These results show that SepSecS mRNA stability in neurons treated with 10^−7^ mol/L of Se was considerably increased and mRNA stability in cells treated with 10^−5^ mol/L of Se was significantly reduced.

## Discussion

As an essential trace element, Se plays an important role in the maintenance of proper nervous system function [3], [5]–[6] and is needed during brain development and metabolism [6], [18], [25]. Consequently, its content in the brain is maintained for prolonged periods of selenium deficiency to a much greater degree than that in other body compartments, which suggests an especially important functions of selenium in this organ [26]. Recent evidence indicates that Se supplementation is necessary for poultry growth performance [27]–[28]. However, the pattern of Se in the avian brain has remained obscure. In this study, we observed Se content in the brain tissues of chickens fed Se-supplemented diets. The results of the present work confirm that Se content in the avian brain is not high in contrast to the whole blood and the gastrointestinal tract tissues [19], and remains remarkably stable during Se supplementation, with other organs most likely accumulating the excess. It suggests that Se is retained within the brain even during Se supplementation, implying a potential importance of the micronutrient in the maintenance of avian brain function.

The physiological functions of Se are thought to result from its existence in a number of selenoproteins, in which Se is present as the amino-acid Sec. Sec is the 21^st^ amino acid, lacks its own tRNA synthetase and is the only amino acid that is synthesized on the cognate tRNA in all domains of life [29]. The synthesis of Sec and its insertion into polypeptides require a complex molecular machinery that recodes in-frame UGA codons, which normally function as stop signals, to serve as Sec codons. During translation of selenoprotein mRNAs, UGA is recoded by the interaction of a specialized elongation factor, EFsec in eukaryotes, with a downstream Sec-insertion sequence element (SECIS). It is incorporated into the amino acid sequence of selenoproteins during translation and is formed by the tRNA-dependent conversion of serine [13], [30]. The biosynthesis and transformation of Sec in eukaryotes is shown in Fig. 1. However, in bacteria Ser-tRNA^Sec^ is directly transformed to Sec-tRNA^Sec^ in a pyridoxal-5′-phosphate-dependent reaction by Sec synthase, the SelA gene product [14]. The occurrence of SepSecS exclusively in selenoprotein-containing organisms may be the most characteristic feature of Sec in eukaryotes with Sec machinery. This suggests that SepSecS is critical for the biosynthesis and transformation of Sec in eukaryotes, and plays an important role in the biological function and metabolism of Se through its regulation of selenoprotein synthesis. However, how it is regulated by Se at the level of SepSecS transcription, which mediate the biological effects of Se, is not known. With the exception of SECIS-binding protein 2 (SBP2) and SECp43 [31], brain expression levels for Sec machinery are not available. In our previous study, we demonstrated that SepSecS is expressed widely in chicken tissues, including neural tissues. In this study, we observed SepSecS mRNA expression levels in the chicken brain tissues and chicken embryos neurons. The results of the present work confirm that SepSecS gene expression in the avian brain is sensitive to dietary Se content. Of note, SepSecS mRNA levels remained stable in neurons treated with 10^−9^–10^−6^ mol/L of Se for 0–48 h (Fig. 5). It is surprising that the SepSecS transcript levels are different between *in vivo* and *in vitro* data concerning the dose-dependent effect of Se. Brain tissues are mainly composed of neurons and glial cells. We hypothesize that the SepSecS mRNA levels in brain cells could present various response patterns to Se, especially between neurons and glial cells. SepSecS gene expression in glial cells could be more sensitive to Se content than neurons. These results suggest that Se affects the mRNA stability of SepSecS in neurons rather than gene transcription, and that SepSecS plays an important role in the avian brain function. However, a time-dependent decrease of SepSecS mRNA levels was observed in neurons treated with 10^−5^ mol/L of Se. This confirms that high concentrations of Se had cytotoxic effects in chick embryo neurons, consistent with the results of the cell viability.

Previous studies suggest that neurons are the primary sites of selenoprotein expression in the brain [31]. We also investigated the underlying mechanisms of regulation in the SepSecS transcription of neurons in response to Se. In the present study, the SepSecS mRNA half-life in chick embryo neurons was measured in the presence or absence of ActD. Relatively low concentrations of Se enhanced the SepSecS mRNA half-life in chick embryo neurons. In contrast, high concentrations of Se significantly reduced the SepSecS mRNA half-life in neuronal cells. These data suggested that Se may alter SepSecS mRNA stability in cells. In addition to transcription regulation, it is apparent that post-transcriptional control of gene expression through mRNA transcript stability is important in the regulation of genes. The mRNA stability is a highly regulated post-transcriptional step tightly coordinated with mRNA translation [32]. The results of this study strongly suggest that the alteration of post-transcriptional stabilization of SepSecS mRNA is an important mechanism of Se-induced elevation and reduction of SepSecS expression levels in the avian brain.

Sec-containing proteins, designated selenoproteins, are likely to account for many of the health benefits derived from Se, and play a pivotal role in the brain. Though the brain does not carry the highest content of Se, in addition to the testis the brain is a privileged organ with a high priority for selenium uptake under low nutritional selenium supplies [26]. Surprisingly, the results of our study found that Se homeostasis in avian brain tissues were preferentially sustained even under conditions of dietary Se supplement. However, how intake of the micronutrient Se affects gene expression and how genetic factors influence Se metabolism in the brain is obscure. The preferential homeostasis of Se in the central nervous system suggests a specific Se uptake mechanism and storage in this organ, and selenoprotein is thought to be a key factor in Se supply and storage [33]–[35]. Importantly, the expression of selenoproteins in the brain depends on the supply. Evidence from the selenoproteome distribution in the mouse brain has indicated that six selenoprotein genes (GPx4, SelK, SelM, SelP, SelW, and Sep15) were expressed at a high level in brain regions [31]. Moreover, this transgenic model provided a direct clue that the hierarchical supply of Se to the brain depends on SelP and the expression of selenoproteins is controlled individually and tissue-specifically by the Se levels [4], [36]. In our previous study, we found that SelW gene expression in the avian neural tissues is sensitive to dietary Se content [20]. Therefore, selenoprotein not only functions as a Se transport molecule, but also within the brain as an extracellular readily-available and reversible Se reservoir [37]. Recent studies suggest that selenoprotein mRNA is degraded by a nonsense-mediated mRNA decay mechanism when Sec is not available for translation [38]–[39]. In this study, we found that Se altered SepSecS mRNA stability and expression in the avian brain. We hypothesize that SepSecS plays an important role on the regulation of Se homeostasis in the avian brain. However, the detailed mechanisms through which Se regulates it's metabolism in the brain tissues of chickens remain unknown.

In summary, the results of this study open new opportunities for research on Se and are important for understanding the relationship between SecSepS, a Sec machinery factor, and homeostasis of Se in the brain. More specifically, this study has led to some initial insights into the mechanisms which establish the hierarchical supply into and retention of Se within the avian brain. Therefore, in the future, selenoproteome distribution in the chicken brain should been determined under conditions of Se-supplement diet conditions.

## Materials and Methods

### Ethics Statement

All chicken experiments were approved by the Institutional Animal Care and Use Committee of Northeast Agricultural University, under the approved protocol number SRM-06.

### Birds and diets

Fifty male chickens (1-day-old; Weiwei Co. Ltd., Harbin, China) were divided into five groups (10 chickens/group) and fed either the commercial granulated diet or the Se-supplemented granulated diet containing 1.0, 2.0, 3.0 or 5.0 mg/kg sodium selenite for 90 days. The basal commercial granulated diet was shown by analysis to contain 0.15 mg/kg Se. On the 90th day of the experiment, all of the chickens were fasted overnight. Following euthanasia with sodium pentobarbital, brain tissue was quickly removed, blotted and then rinsed with ice-cold sterilized deionized water, frozen immediately in liquid nitrogen and stored at −80°C until it was processed. Se content and mRNA levels of SepSecS were measured.

### Preparation of chick embryo neurons

Primary cultures of chick embryo neurons were prepared according to a modified Mangoura and Dawson protocol [21]. In brief, Cultures were prepared by dissociating 8-day-old chick embryo cerebral hemispheres using 6-well plates for cell culture and turning experiments. To achieve optimum growth conditions, plates were coated overnight with 0.1 mg/mL poly-D-lysine (Sigma, USA). The cerebral hemispheres were dissected aseptically, cleaned of adhering meningeal membranes and blood vessels, and dissociated by trypsin treatment. Cell suspension was prepared by gently pushing the splenic pulp through a sterile stainless steel mesh with a pore size of 100 µM. The dispersed cells were plated initially in complete cell culture medium (DMEM containing HEPES and 2 mM glutamine, supplemented with 10% fetal bovine calf serum and antibiotics) for 24 h. To purify chick embryo neurons, the culture medium was supplemented with 2 µg/mL cytarabine (Sigma, USA). Following culture for 24 h, medium was removed from the cultures and the cell monolayers were washed three times with Hank's balanced salt solution. Then, neurons were incubated in complete cell culture medium.

### Neuron cultures and treatments

Neurons were seeded into six-well plates precoated with poly-D-lysine at a density of 1.0×10^6^ viable cells per well in 2 mL of complete medium. All cultures were incubated at 37°C in an atmosphere of 5% CO_2_ and 95% air, saturated with water. After an attachment period of 48 h, cell monolayers were washed twice with Hank's balanced salt solution. Then, neurons were grown in 2 mL of fresh complete medium and treated with 0 mol/L, 10^−9^ mol/L, 10^−8^ mol/L, 10^−7^ mol/L, 10^−6^ mol/L or 10^−5^ mol/L of Se as sodium selenite (Sigma, USA) for 0 h, 3 h, 6 h, 12 h, 24 h or 48 h, respectively. Neurons were harvested for analysis of SepSecS mRNA level. Each treatment was done in six wells in three separate experiments.

### Determination of the survival and morphology of Neurons

#### WST-8 assay

To assess the survival activity of neurons, we performed a WST-8 reduction assay with a kit (Beyotime Inst Biotech, China) according to the manufacturer's instructions. WST-8 was added to the culture for 3 h at 37°C prior to colorimetry. The percentage of WST-8 activity was represented as the ratio of activity at different time points to that in the beginning of culture.

#### LDH release assay

Neuron damage was quantified by measuring the activity of cytosolic LDH released into the culture medium from damaged cells, by using a LDH Detection Kit (Nanjing Jiancheng Bioengineering Institute, China) according to the manufacturer's instructions. Enzyme activity was evaluated by colorimetry.

#### Trypan blue exclusion test

Neuronal cell death was assessed by with a trypan blue exclusion test. Neurons were harvested and stained with 0.4% trypan blue. Stained cells were evaluated as dead, and results were represented by the percentage of dead cells among the total cells.

#### Neuron morphometric measurement

The morphology of treated and untreated neurons was visualized under light microscopy (Eclipse-*Ti*, Nikon, Japan) at 400× magnification. Under a light microscope, digital images were taken from 10 randomly selected fields which contained more than 20 cells. Neurite lengths on the digitized images were measured using Image J, an image analysis system (NIH Image J software located at http://rsb.info.nih.gov/ij/). The clusters of cells were excluded from the measurement of neurite length.

### Determination of Se concentration in tissues

Se content in the tissues and whole blood was estimated using a method described by Hasunuma et al [22]. The assay is based on the principle that the Se contained in samples is converted to selenous acid in response to acid digestion. The reaction between selenous acid and aromatic-o-diamines, such as 2,3-diamino-naphthalene (DAN), leads to the formation of 4,5-benzopiazselenol, which displays a brilliant lime-green fluorescence when excited at 366 nm in cyclohexane. Fluorescence emission in extracted cyclohexane was measured by a fluorescence spectrophotometer.

### Determination of SepSecS mRNA levels by quantitative RT-PCR

Total RNA was isolated from the tissue samples (50 mg tissue; n = 3/diet group) and the neuron monolayers using TRIZOL (Invitrogen, China) and according to the manufacturer's protocol. Dried RNA pellets were resuspended in 40 µL of diethyl-pyrocarbonate-treated water. The concentration and purity of total RNA were determined spectrophotometrically at 260/280 nm. First-strand cDNA was synthesized from 2 µg of total RNA using oligo dT primers and Superscript II reverse transcriptase according to the manufacturer's instructions (Invitrogen, China). Synthesized cDNA was diluted five times with sterile water and stored at −80°C before use.

Primer Premier Software (PREMIER Biosoft International, USA) was used to design specific primers for SepSecS and GADPH based on known chicken sequences (Table 1). Quantitative real-time PCR was performed on an ABI PRISM 7500 Detection System (Applied Biosystems, USA). Reactions were performed in a 20 µL reaction mixture containing 10 µL of 2× SYBR Green I PCR Master Mix (TaKaRa, China), 2 µL of either diluted cDNA, 0.4 µL of each primer (10 µM), 0.4 µL of 50× ROX reference Dye II and 6.8 µL of PCR-grade water. The PCR procedure for SelW and GADPH consisted of 95°C for 30 seconds followed by 40 cycles of 95°C for 15 seconds, 60°C for 30 seconds and 60°C for 30 seconds. The melting curve analysis showed only one peak for each PCR product. Electrophoresis was performed with the PCR products to verify primer specificity and product purity. A dissociation curve was run for each plate to confirm the production of a single product. The amplification efficiency for each gene was determined using the DART-PCR program [23]. The mRNA relative abundance was calculated according to the Pfaffl method [24], accounting for gene-specific efficiencies and was normalized to the mean expression of GADPH.

**Table 1:** Primers used for quantitative real-time PCR.

Target gene	GenBank accession no	Primer	Sequence (5′–3′)	PCR fragment length
SepSecS	NM_001031158.1	Forward	5′- CATGAACTTGCCATAATGGAC -3′	112 bp
		Reverse	5′- GGATCAACCTATAGTGCCTT -3′	
GADPH	K01458	Forward	5′-AGAACATCATCCCAGCGT-3′	182 bp
		Reverse	5′-AGCCTTCACTACCCTCTTG-3′	

### Determination of SepSecS mRNA half-life

The mRNA half-life determinations were performed as described previously [40]. Briefly, the control and Se-treated neurons were further incubated with 5 µg/mL actinomycin D (ActD, Sigma, USA) for 0 h, 3 h, 6 h, 9 h, 12 h, 24 h or 48 h at which time points RNAs were extracted and the level of SepSecS mRNA was measured by quantitative RT-PCR, as described above. Cell transcription was stopped by the addition of the RNA polymerase inhibitor ActD. The mRNA half-life was extrapolated from the SepSecS mRNA decay curve as the time point after ActD treatment at which there was 50% of the initial mRNA level remaining.

### Statistical analysis

Statistical analysis of Se concentrations and mRNA levels was performed using SPSS statistical software for Windows (version 13; SPSS Inc., Chicago, IL, USA). When a significant value (*P*<0.05) was obtained by one-way analysis of variance, further analysis was done. All data showed a normal distribution and passed equal variance testing. Differences between means were assessed by Tukey's honestly significant difference test for post hoc multiple comparisons. Data are expressed as mean ± standard deviation. Differences were considered to be significant at *P*<0.05.

## Supporting Information

Figure S1 **Effects of Se on the morphology of chicken embryo neurons.** The chicken embryo neuron monolayers were treated with 0 mol/L, 10^−9^ mol/L, 10^−8^ mol/L, 10^−7^ mol/L, 10^−6^ mol/L or 10^−5^ mol/L of Se as sodium selenite for 0 h, 3 h, 6 h, 12 h, 24 h or 48 h, respectively. The morphology of treated and untreated neurons was visualized under the light microscopy (magnification: ×400, Bar = 50 µm). Note the neurite length, branches, and the morphological alterations of neurons [41].
(TIF)

Figure S2 **Effects of Se on neurite outgrowth in chicken embryo neurons.** (A) Population analysis of neurite bearing cells. (B) Average neurite lengths. (C) Population analysis of neurite branches. Bars represent mean ± standard deviation of triplicate cultures. Bars with “*” are significantly different from controls as measured by one-way analysis of variance followed by a Tukey's multiple comparison test (* *P*<0.05, ** *P*<0.01, *** *P*<0.001) [41].
(TIF)
