# Supplementary figures and images for: Priority in Selenium Homeostasis Involves Regulation of SepSecS Transcription in the Chicken Brain
Source: PLoS One. 2012 Apr 20;7(4):e35761. doi: 10.1371/journal.pone.0035761 (PMC3334997; doi:10.1371/journal.pone.0035761)

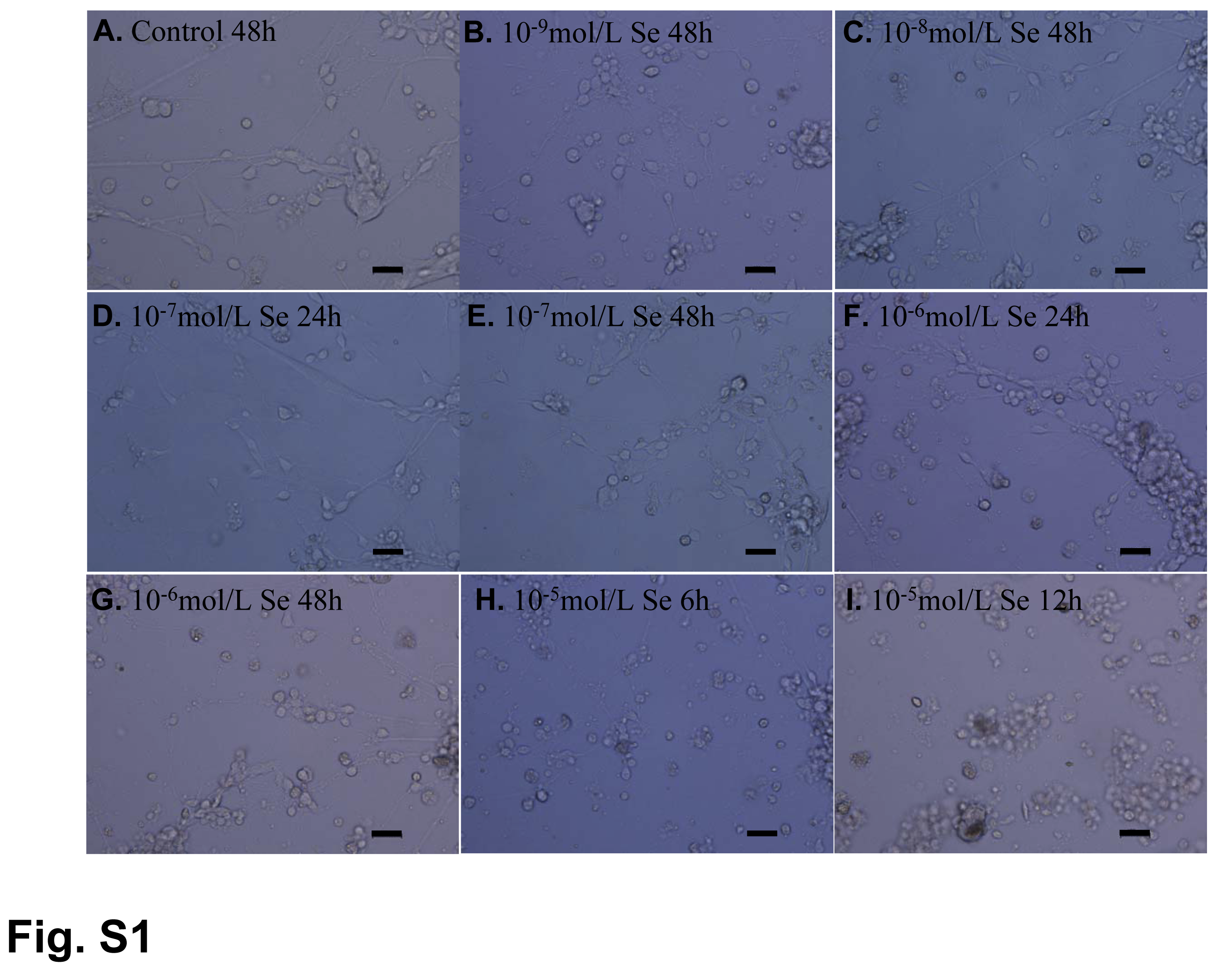

Supplement: Figure S1 — Effects of Se on the morphology of chicken embryo neurons. The chicken embryo neuron monolayers were treated with 0 mol/L, 10−9 mol/L, 10−8 mol/L, 10−7 mol/L, 10−6 mol/L or 10−5 mol/L of Se as sodium selenite for 0 h, 3 h, 6 h, 12 h, 24 h or 48 h, respectively. The morphology of treated and untreated neurons was visualized under the light microscopy (magnification: ×400, Bar = 50 µm). Note the neurite length, branches, and the morphological alterations of neurons [41]. (TIF) [file pone.0035761.s001.tif]

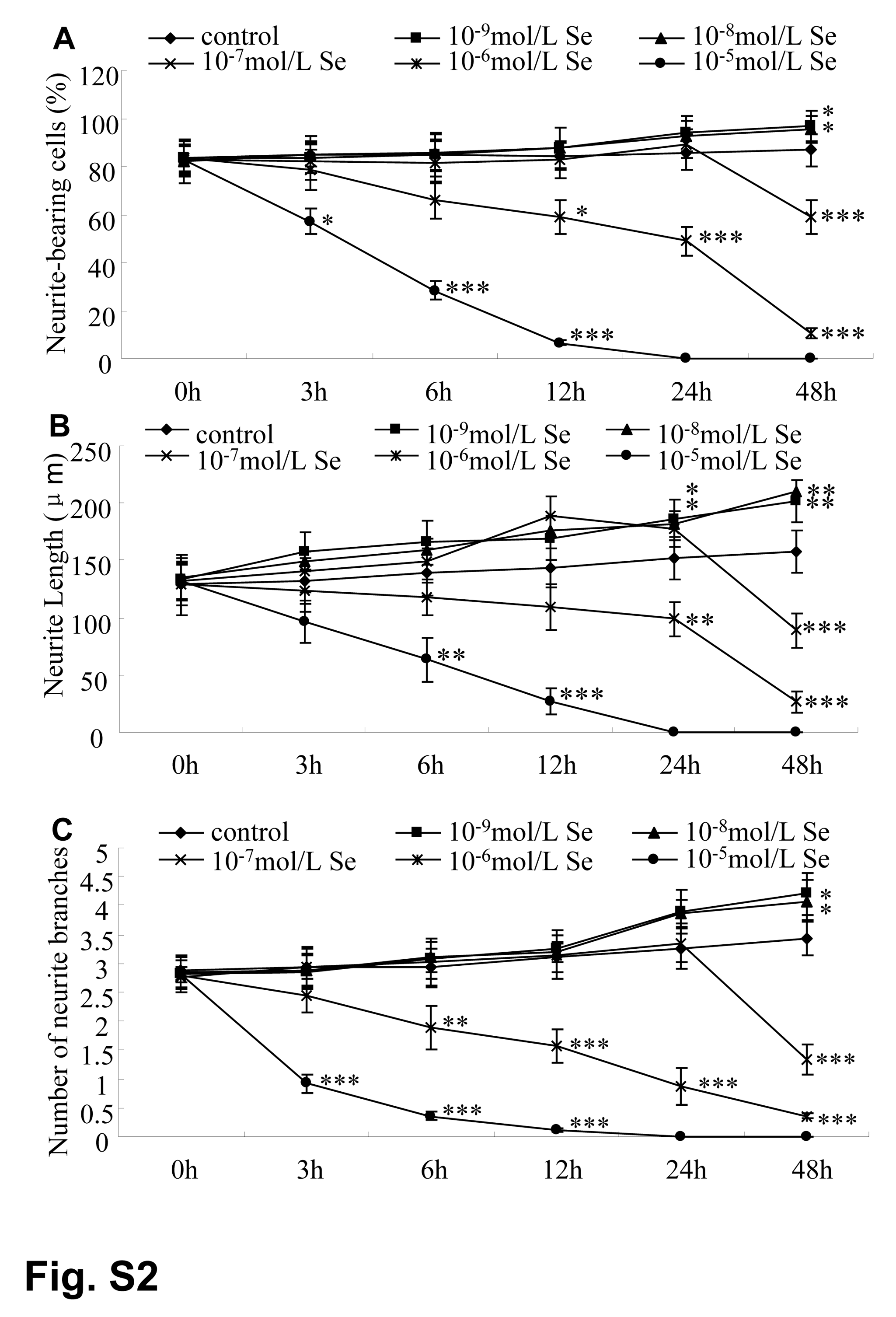

Supplement: Figure S2 — Effects of Se on neurite outgrowth in chicken embryo neurons. (A) Population analysis of neurite bearing cells. (B) Average neurite lengths. (C) Population analysis of neurite branches. Bars represent mean ± standard deviation of triplicate cultures. Bars with “*” are significantly different from controls as measured by one-way analysis of variance followed by a Tukey's multiple comparison test (* P<0.05, ** P<0.01, *** P<0.001) [41]. (TIF) [file pone.0035761.s002.tif]
